# Upward trends of syphilis in the non-pregnant adults: A six-year report on clinical and epidemiological profile of syphilis from a tertiary care center, India

**DOI:** 10.3389/fpubh.2022.908591

**Published:** 2022-07-26

**Authors:** Dhanalakshmi Solaimalai, Ankan Gupta, Leni George, Abi Manesh, Rajiv Karthik, Dharshini Sathishkumar, C. V Dincy Peter, George M Varghese, Susanne A Pulimood, Rajesh Kannangai, John AJ Prakash

**Affiliations:** ^1^Department of Clinical Microbiology, Christian Medical College, Vellore, India; ^2^Department of Dermatology, Christian Medical College, Vellore, India; ^3^Department of Infectious Diseases, Christian Medical College, Vellore, India; ^4^Department of Clinical Virology, Christian Medical College, Vellore, India

**Keywords:** syphilis, STI, MSM, HIV, neurosyphilis, seroprevalence

## Abstract

Since 2000, a resurgence of syphilis has been noted in many developed and developing countries, especially among men who have sex with men (MSM). Incidence and prevalence of syphilis in pregnant women have been reduced drastically by mandatory screening in early pregnancy. Insufficient data in other populations especially from developing countries limit targeted public health interventions. This study aimed to describe the clinical and epidemiological profile of serologically confirmed syphilis cases among the non-pregnant high-risk group reporting to a tertiary care center in Southern India. A retrospective study was carried out in a tertiary care center in Southern India for 6 years from 2015 to 2020. A total of 265 serologically confirmed syphilis patients were included. A statistically significant increase in positivity from 0.52 to 2.1% was observed in this study (2015 to 2020). Among risk factors, high-risk behavior with multiple heterosexual partners was the commonest (51.3%), followed by marital partners who tested positive (9.4%) and MSM (7.5%). The majority of the patients were diagnosed at the latent stage (79%), followed by secondary syphilis (10%) and tertiary syphilis (8%). A quarter of patients (23%) were coinfected with HIV. Serological non-responsiveness was more common among HIV infected (47 vs. 24%). Sixteen had neurosyphilis and six had ocular involvement. HIV co-infection complicated 50% (8/16) of neurosyphilis patients. Syphilis is still prevalent, especially in high-risk groups including those are attending STI clinics. Further prospective multicentric studies are needed to identify and implement public health measures.

## Introduction

Syphilis is one of the four curable and preventable sexually transmitted infections (STI) apart from chlamydia, trichomoniasis, and gonorrhea. The incidence and prevalence of syphilis had decreased after the introduction of the HIV prevention program. However, the increased prevalence has been noted in many developing countries and western countries, especially among specific subgroups since 2000 (1). The recent systematic analysis showed a very high pooled global prevalence of 7.5% (2000 to 2020) among men who have sex with men (MSM) compared to 0.5% among men in the general population estimated in 2016 (2, 3). The Chinese notifiable infectious diseases surveillance registry reported a three-fold increase in syphilis cases over 10 years (from 135,210 in 2005 to 441,818 in 2014) (4).

The WHO periodically releases the global estimates of four curable STI, providing evidence for policymakers to monitor, evaluate and improve STI prevention programmes. According to that, more than one million new curable STI are acquired every day worldwide and 7.1 million new cases of syphilis were estimated in 2020 (5). The WHO released a global health sector strategy for 2016 to 2021, with a goal of a 90% reduction in syphilis incidence worldwide and 50 or fewer cases of congenital syphilis per 100,000 live births in 80% of countries (6). This prioritized eliminating congenital syphilis by implementing mandatory screening and treating syphilis among pregnant women.

The National AIDS Control Organization (NACO) in India launched a national strategy for the elimination of parent-to-child transmission of syphilis in February 2015 based on the global initiative by WHO in 2007. To eliminate parent-to-child transmission of syphilis and HIV by 2020, the Government of India has taken a policy of universal screening of pregnant women for syphilis and HIV during the first visit (1st trimester) as part of the essential antenatal care package. Testing is done at all levels of healthcare facilities such as medical colleges, district hospitals, primary health centers and subcentres at free of cost (7, 8). At a tertiary care center in north Tamil Nadu, a reduced seroprevalence rate among pregnant women from 0.4% (1998-99) to <0.1% (2011-15) was observed due to an effective intervention after mandatory screening in the early pregnancy (9, 10). However, there is no clear epidemiological data to support specific subgroup screening from India (11). Therefore, this study aimed to describe clinical and epidemiological profiles of serologically confirmed syphilis cases among the non-pregnant high-risk group reporting to a tertiary care center in Southern India.

## Materials and Methods

We conducted a retrospective study in our 2600-bed tertiary care center spanning 6 years (from January 2015 to December 2020). The Clinical Microbiology laboratory is a large volume laboratory (ISO 15189: 2012 accredited) that receives approximately 20,000 samples per year for syphilis serology from the following groups of individuals: antenatal women, neonates of suspected congenital syphilis, PLHIV patients before starting cART, patients with suspicion of syphilis based on skin or genital rash and patients seeking STI diagnosis and treatment.

For this study, we included adult non-pregnant patients (>18 years) with serologically confirmed syphilis which is defined as Venereal Disease Research Laboratory (VDRL) reactive and *Treponema pallidum* haemagglutination (TPHA) positive or TPHA alone positive. The VDRL assay was performed using the VDRL antigen (Institute of Serology, Calcutta, India) as described previously (9). The TPHA assay (Omega Diagnostics, Scotland, UK) was performed and interpreted according to the manufacturer's instructions. In our center, the traditional algorithm is followed for the screening of syphilis. First, the patient is screened with a VDRL test. If VDRL is reactive then confirmed with TPHA (specific test). However, both the tests were performed simultaneously for the patients who came to our center for confirmation of syphilis (treated outside).

Pregnant women and children (<17 years) were excluded from the study. The following clinical and epidemiological data for each patient were recorded from the patients' electronic medical records: risk factors, clinical presentation, stage of the disease, treatment, follow-up details, other associated STIs, and cerebrospinal fluid (CSF) parameters for neurosyphilis cases.

The stage of the disease at diagnosis was assigned by the treating clinicians as per the standard criteria (12). Neurosyphilis cases were diagnosed by the combination of serological tests for syphilis plus CSF analysis of elevated cells >5/mm^3^ with lymphocytic predominance with or without elevated protein (>45 mg/dL). The symptomatic patients were classified into early (meningitis, meningo-vascular, neuro-ocular, ocular) and late (general paralysis of insane) neurosyphilis based on the clinical presentation, CSF parameters, eye examination and MRI brain by Infectious Disease Physicians. Asymptomatic cases were diagnosed based on CSF analysis that was performed for pre-ART/ co-infection work-up among HIV-infected patients (13).

Serological response to the treatment was defined as a four-fold reduction in VDRL titer between the initial titer and subsequent testing. The time interval of retesting is at 6, 12 and 24 months on those reporting for follow-up. A fourfold reduction in titer at any time during the 24 months of follow-up is considered as definitive evidence of cure (12). The old infection was not counted if the patient came for follow-up.

Data were summarized using mean and standard deviation (SD) for continuous variables and frequency along with percentage for categorical variables. All categorical associations were tested using chi-square statistics. The analysis was done using Microsoft Excel and a *p*-value <0.05 was considered statistically significant.

Our Institutional Review Board and the ethical committee approved the study (IRB Min No 13417, dated 23.9.2020).

## Results

During the study period, a total of 1,12,689 samples were tested for syphilis serology. Among these 86,691 (77%) samples were collected from antenatal mothers and 25,998 (23%) from other patients. Totally 265 non-pregnant patients satisfied the inclusion criteria and were analyzed. The demographic details of the study patients are described in Table 1. Male preponderance was noted (218/265, 82.3%). The year-wise analysis of mean age was performed and observed that slight decrease in mean age from 38.5 in 2015 to 35 in 2020 (Table 1).

**Table 1:** The demographic details.

**Parameters**	**Observation**	
**Male/female ratio**	**4.63 (218/47)**	
**Married/ unmarried**	**3.5 (206/59)**	
**Mean age (SD)**	**36 (±10.2)**	
**Annual mean age and sex ratio** 2015 2016 2017 2018 2019 2020	**Mean age** 38.5 39 36.4 35 34.5 35	**Male/female ratio** 2.4 (19/8) 5.4 (27/5) 4.6 (37/8) 2.9 (40/14) 10.7 (64/6) 5.2 (31/6)
**Age-wise distribution and sex ratio** 17 to 24 25 to 34 35 to 44 45 to 54 55 to 64 >65	***n*** (%) 29 (11%) 109 (41%) 74 (28%) 37 (14%) 12 (4.5%) 4 (1.5%)	**Male/female ratio** 2.6 (21/8) 5.4 (92/17) 5.2 (62/12) 4.3 (30/7) 5 (10/2) 3 (3/1)
**Year-wise positivity** 2015 2016 2017 2018 2019 2020	**Number of samples tested** 5169 5291 5628 4390 3756 1764	**Positivity *n*(%)** 27 (0.52%) 32 (0.6%) 45 (0.8%) 54 (1.2%) 70 (1.86%) 37 (2.1%)
**Risk factors** Multiple sex partners Without HIV PLHIV MSM Without HIV PLHIV Partner tested positive Denied of any HRB Data not available	***n*** (%) 136 (51.3%) 84 (61.8%) 52 (38.2%) 20 (7.5%) 11 (55%) 9 (45%) 25 (9.4%) 48 (18%) 36 (13.6%)	
**Associated STIs and other infections** HIV *Herpes simplex virus* *Hepatitis B virus* *Hepatitis C virus* *Neisseria gonorrhea*	***n*** (%) 61 (23%) 6 (2.3%) 6 (2.3%) 2 (0.8%) 1 (0.4%)	

PLHIV, People living with human immunodeficiency virus; MSM, Men have sex with men; HRB, high risk behavior.

The confirmed cases increased gradually from 2015 to 2019. We observed a sudden increase in the number of cases in the year 2019. There was a decline in 2020 due to the COVID 19 pandemic and a smaller number of samples tested during that period. However, the positivity increased from 1.86% in 2019 to 2.1% in 2020. The pooled percentage of patients who presented with genital ulcers or other genital symptoms prior to 2020 (2015 to 2019) was 18% (41/228) compared to13.5% (5/37) in 2020. This reflects the true increase in positivity, though samples tested were less during the COVID 19 pandemic.

**Figure 1:**
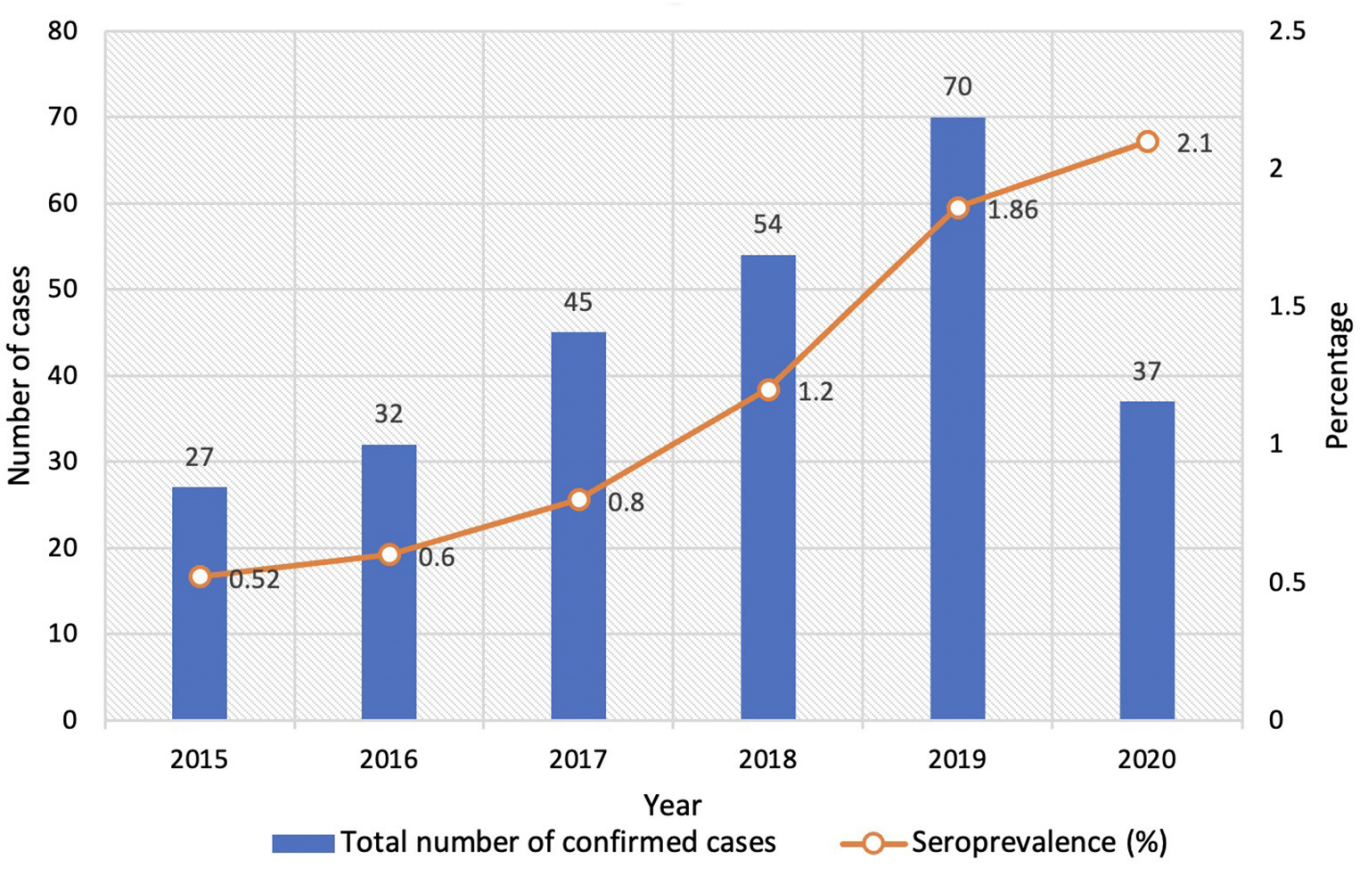
Year-wise distribution of cases and seroprevalence.

The samples tested during the study period (2015 to 2020) were 5,169, 5,291, 5,628, 4,390, 3,756, and 1,764, respectively. The year-wise distribution of confirmed cases and seroprevalence is shown in Figure 1. A statistically significant increase in positivity from 0.52 to 2.1% was observed in this study (*p* = 0.0015).

Amongst the risk factors, a history of multiple sex partners (heterosexual) was the most common risk factor (51.3%), followed by marital partners who tested positive (9.4%) and MSM (7.5%) (Table 1).

## Stage of the Disease

**Figure 2:**
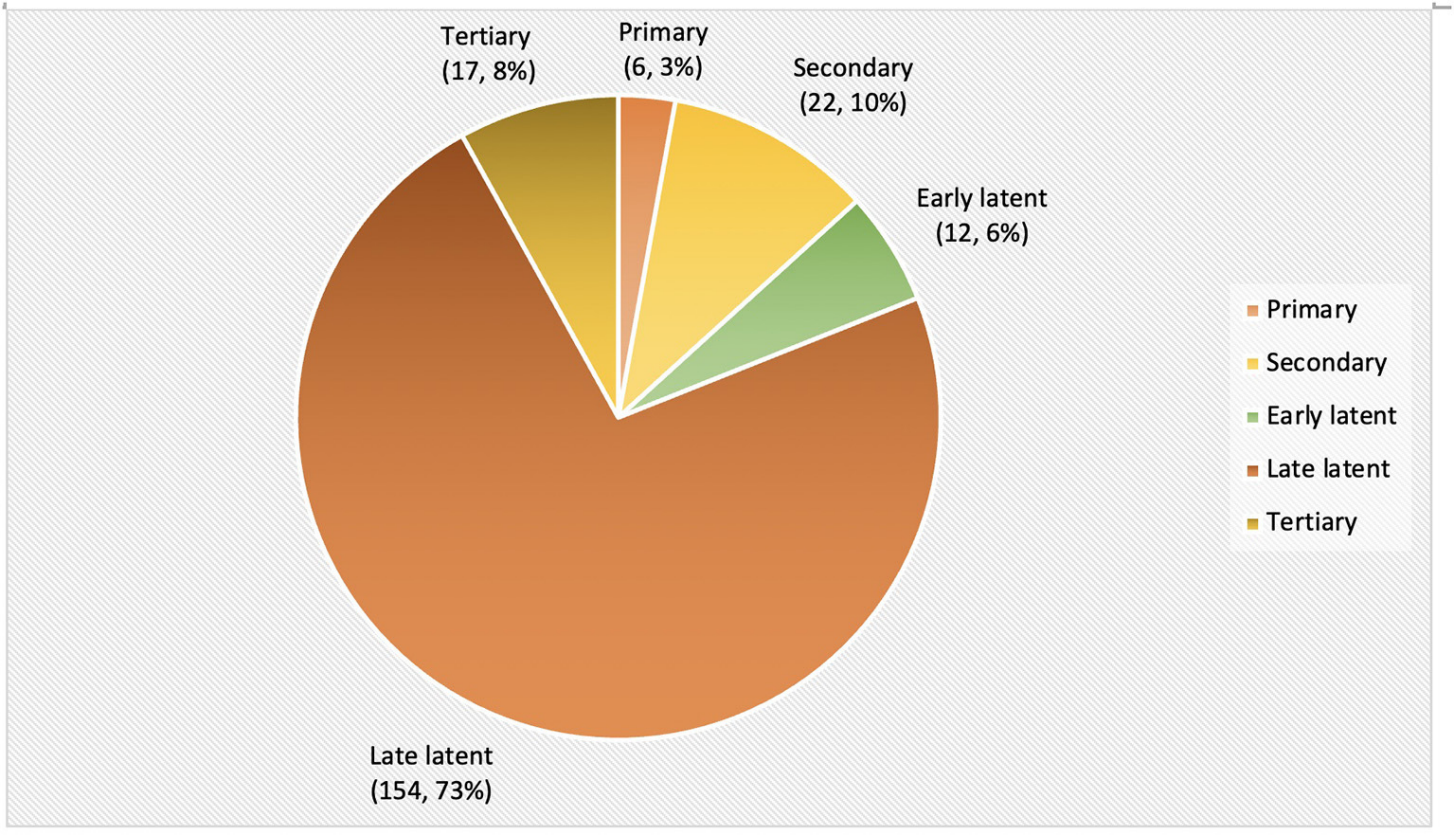
The stage of syphilis at diagnosis.

Among 265 patients, 54 were partially treated elsewhere before presentation in our center. These 54 patients were excluded for further analysis due to insufficient data regarding the stage of disease at diagnosis and treatment history. The stage of the disease at the time of diagnosis is illustrated (Figure 2) for 211 remaining patients. The majority of the patients were diagnosed at the latent stage (166, 79%), followed by secondary syphilis (22, 10%) and tertiary syphilis (17, 8%).

**Figure 3:**
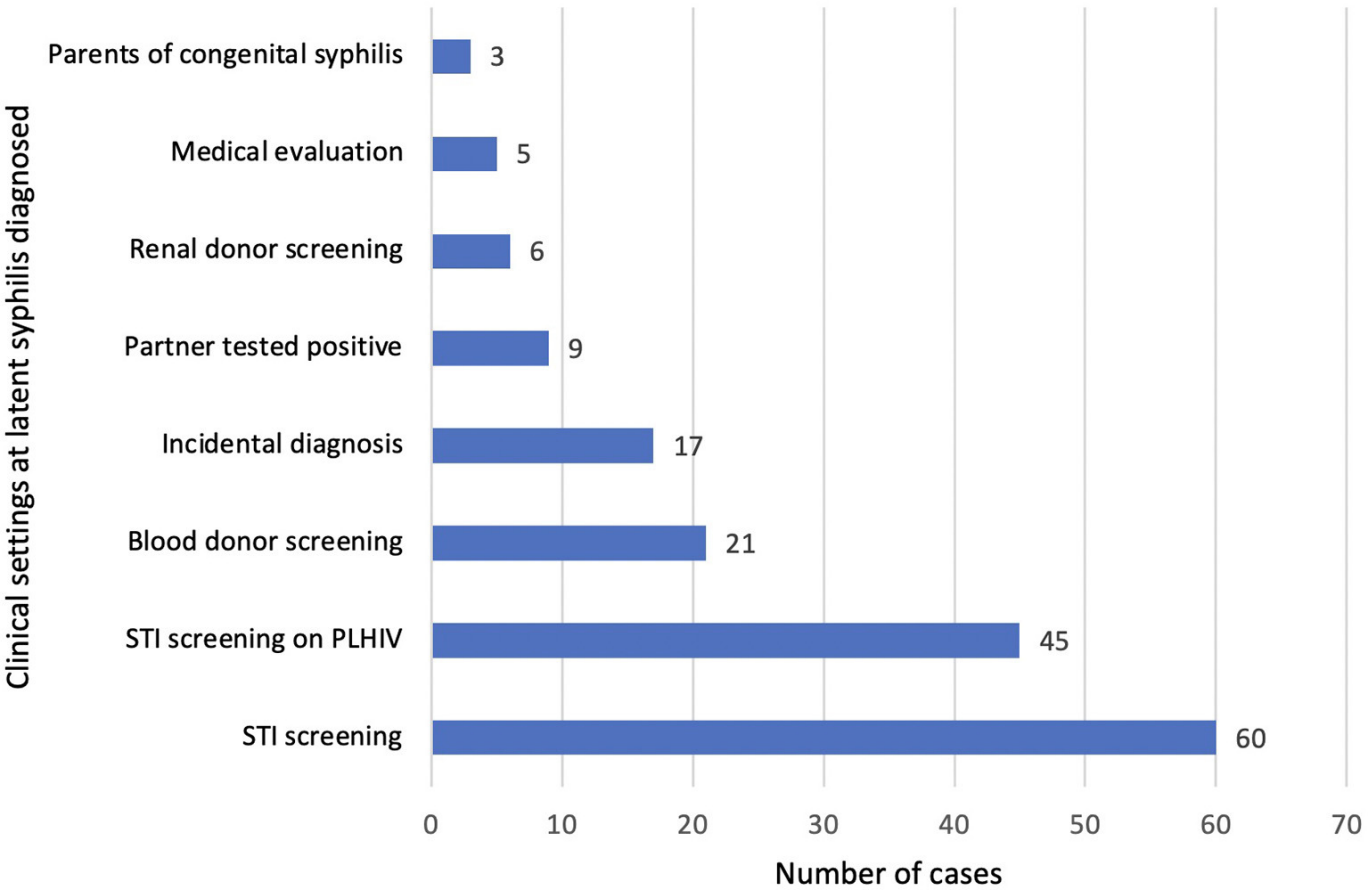
Diagnosis of latent syphilis.

We looked at the various clinical settings where latent syphilis was diagnosed (Figure 3). The latent syphilis cases were diagnosed most commonly during an active screening among those presenting with symptoms suggestive of STI with or without a high-risk behavior (*n* = 60) and PLHIV (*n* = 45). Passive screening among blood donors, organ donors and medical evaluation (for a foreign work visa) identified 32 patients (Figure 3).

During the study period, a total of 17 patients were diagnosed with tertiary syphilis. Sixteen patients were diagnosed with neurosyphilis and one with gummatous syphilis. Neuro-ocular syphilis (*n* = 6) was the most common, followed by asymptomatic neurosyphilis and meningeal (*n* = 4 each), and general paresis of insane *(n* = 2). CSF analysis showed a characteristic pleocytosis with lymphocyte predominance and elevated protein among all early neurosyphilis cases. However, CSF analysis was normal in late neurosyphilis cases (general paresis of insane) (Table 2). HIV co-infection was identified in 50% (8/16) of patients. All except two patients were treated either with intravenous aqueous crystalline penicillin G or Inj. Ceftriaxone for 2 weeks. Nine out of 16 patients had at least one follow-up visit with us after treatment and five among them showed serological response i.e., either a four-fold reduction in titer or non-reactive serum VDRL.

**Table 2:** Serum and CSF parameters of neurosyphilis patients.

	**Early neurosyphilis**				**Late neurosyphilis**
**Total cases (*n* = 16)**	**Meningeal/ meningo-vascular neurosyphilis** **(*n* = 4)**	**Asymptomatic neuro syphilis** **(*n* = 4)**	**Neuro-ocular syphilis** **(*n* = 4)**	**Ocular syphilis** **(*n* = 2)**	**general paresis of insane** **(*n* = 2)**
Mean age	37	39	39	37	52
Gender male *n*(%)	2 (50)	3 (75)	4 (100)	0	2 (100)
HIV positive *n*(%)	3 (75)	4 (100)	1 (25)	0	0
Serum TPHA positive - *n*(%)	4 (100)	4 (100)	4 (100)	2(100)	2 (100)
Serum VDRL reactive - *n*(%)	4 (100)	4 (100)	3 (75)	0	1 (50)
CSF VDRL/TPHA reactive - *n*(%)	3 (75)	3 (75)	2^*^ (67)	0	1 (50)
CSF WBC >5 cell/mm^3^	3 (75)	3 (75)	3^*^ (100)	0	0
CSF Protein >45 mg/dL -*n* (%)	3 (75)	4 (100)	3^*^ (100)	0	2 (100)

* CSF analysis not done in one patient.

### Treatment and follow-up

In our hospital, 164 (77.7%) out of 211 diagnosed patients were treated appropriately for the stage of the disease with benzathine penicillin and 47 (22.3%) patients lost follow-up after diagnosis.

With the available follow-up data among 52 patients, 7 (13.5%), 16 (30.8%), 10 (19.2%), 6 (11.5%) and 13 (25%) patients had a follow-up visit till 3 months, 6 months, 12 months, 18 months and ≥ 24 months, respectively. Re-infection or second infection was considered in a patient who was treated adequately and had the same or rise in VDRL titer in a follow-up visit. Those patients were treated again and not counted as a new infection in the study. Moreover, very few patients (*n* = 2) were identified as re-infection in this study.

Serological non-response to the treatment was higher among HIV co-infected patients (46.7%) compared to HIV-negative patients (24.3%). However, this is statistically not significant (Table 3).

**Table 3:** Serological response to the treatment.

	**HIV positive**	**HIV negative**	***p*** **-value**
Total number of patients (*n* = 52)	15	37	0.21 (Chi-square statistic = 1.56)
Four-fold reduction of titer observed *n* (%)	8 (53.3%)	28 (75.7%)	

p-value <0.5 is significant.

## Discussion

Syphilis is not uncommon in developing countries despite mandatory testing in pregnant women. There is a paucity of data from other groups (1). We observed a steady increase in syphilis positivity over 6 years in our study group (0.52 to 2.1%). A previous Indian study reported a comparable increase from 0.95 to 1.76% in 6 years (14), while another HIV care clinic-based study demonstrated an increase from 0.7 to 1.3% (15). Given the public health perspective, this finding mandates the need for constant surveillance among high-risk groups.

Amongst the risk factors, a history of multiple sex partners (heterosexual) was the most common risk factor (51.3%), followed by marital partners who tested positive (9.4%) and MSM (7.5%). In western countries, MSM is the most common risk factor (16). Interestingly, evidence of high-risk sexual behavior was unavailable for approximately one-third of our patients. This may be due to the healthcare provider's concern about the patient's objection to the sensitive question regarding sexual behaviors (17). The Centers for Disease Control and Prevention (CDC) and US Preventive Services Task Force (USPSTF) recommend screening of syphilis in asymptomatic non-pregnant adults and adolescents who are at risk. The CDC also recommends more frequent screening at 3 to 6 months intervals in sexually active MSM and at least annually in people with HIV (18, 19).

Early diagnosis and treatment is a must to prevent the transmission and progression of the disease. Our study observed that nearly one-fourth of the diagnosed patients were lost to follow-up from our center. This has implications like disease progression and transmission of disease to the sexual partners. We also observed two untreated latent syphilis patients who progressed to neurosyphilis. This indicates the importance of follow-up and completion of treatment at any stage of the disease (20).

Syphilis and HIV co-infection is viewed as a potentially dangerous combination since both diseases negatively impact treatment response to each other. Previous studies observed serological non-response to syphilis treatment, cognitive impairment, and virological failure of ART among syphilis and HIV co-infected patients (21–23). Our study observed that nearly one-fourth of the study population had co-infection with HIV. The co-infection rates are varied in the literature, ranging from 6.4 to 34% (24–26). Serological non-responsiveness was identified in 47% of HIV patients compared to 24% in non-HIV patients among the available follow-up details in 52 patients, although the finding was not statistically significant.

Neurosyphilis was diagnosed in 13% (8/61) of HIV-co-infected patients. In concordance with other studies, ocular manifestations were the most common presentation among neurosyphilis patients (14, 27). Neuro-ocular syphilis was commonly identified in HIV-negative patients (5/6), similar to a study by Borges et.al (27).

We observed that most of the patients (166/211, 79%) were diagnosed at the latent stage of the disease. This is comparable to a study from China that identified 51.9% of latent syphilis, 9% of primary syphilis, and 19.8% of secondary syphilis for a period of 9 years from 2011 to 2019 (28). In contrast, other studies have demonstrated primary and secondary syphilis as the common presentation among STI clinic attendees (29, 30).

This study has limitations. First, as data was collected retrospectively from the patients' electronic medical records, some subjects had incomplete data for some of the variables. Second, though the patients were from wide geographic areas, the results obtained are from a single tertiary care center. Third, the follow-up of patients after diagnosis and treatment was limited since the hometown of many patients was far from our center. However, this study provides information that syphilis is still prevalent and suggests that there is a rise in syphilis cases amongst those at risk. Further, this is not a random sampling as those with risk factors and reporting to a tertiary care center were tested for syphilis (targeted sampling) and therefore our reports are subject to bias.

Still, the data generated from this study provides information regarding the age group affected and the nature of transmission predominantly among heterosexuals. This will help us make a policy regarding the possible preventive measures to be taken.

In summary, we report in a large hospital-based study, a sustained increase in cases of syphilis among non-pregnant patients over a six-year period. We also show that the drivers are very different in the Indian context - transmission predominantly happens via multiple heterosexual contacts and the contribution of MSM remains low. We also highlight a considerable proportion of our patients are HIV coinfected - highlighting the close association and need for complementary efforts to prevent syphilis in our population. Appropriate management of syphilis according to the stage will reduce morbidity and further transmission.

## Conclusion

This hospital-based study suggests that syphilis is not uncommon. Heterosexual transmission is the commonest risk factor and the PLHIV group is also significantly affected. To adequately control syphilis, first, awareness needs to be increased among the general public about the disease especially its association with HIV. Second, the screening and follow-up of high-risk patients need to be intensified. Healthcare providers must elicit the sexual history to identify the high-risk patients for the screening (17, 31). Moreover, the rapid point of care tests for syphilis will improve the identification of high-risk patients (31). Third, all the diagnosed patients at any stage should be treated appropriately to prevent the transmission and progression of the disease. Healthcare setup should have a system to identify the missing patients for treatment and follow-up.

## Data availability statement

The original contributions presented in the study are included in the article/supplementary material, further inquiries can be directed to the corresponding author/s.

## Ethics statement

The studies involving human participants were reviewed and approved by Institutional Review Board and Ethical Committee, Christian Medical College and Hospital, Vellore, India. Written informed consent for participation was not required for this study in accordance with the national legislation and the institutional requirements.

## Author contributions

DSo, JP, AG, LG, AM, RKar, DSa, CP, GV, SP, and RKan contributed to the concept and design of the study. DSo, JP, AG, LG, AM, RKar, DSa, and CP contributed in data acquisition. DSo drafted the manuscript. DSo and JP involved in analysis. JP, GV, SP, and RKan involved in interpretation. JP contributed in supervision. GV, SP, and RKan involved in data analysis. JP, AG, LG, AM, RKar, DSa, CP, GV, SP, and RKan contributed in critically reviewing the manuscript. All authors contributed to the article and approved the submitted version.

## Conflict of interest

The authors declare that the research was conducted in the absence of any commercial or financial relationships that could be construed as a potential conflict of interest.

## Publisher's note

All claims expressed in this article are solely those of the authors and do not necessarily represent those of their affiliated organizations, or those of the publisher, the editors and the reviewers. Any product that may be evaluated in this article, or claim that may be made by its manufacturer, is not guaranteed or endorsed by the publisher.
